# 

**Published:** 1916-05-20

**Authors:** 


### CONTENTS.

| | PAGE |
|---|---|
| **The Medical Profession: Compulsory or Voluntary Service?** ... ... ... ... ... | 161 |
| **Diagnosis and Personality** ... ... ... ... | 162 |
| **Hospital and Institutional News** ... ... ... | 163 |
| Sir Perceval Nairne and his Portrait—The Bishop's Last—Nervous Breakdown in the Navy—Wittenberg's Heroic Doctors—Three New Benevolent Societies—A "Rara Avis": The Medical "C.O."—Economy in the Annual Report—Surcharging of Staffordshire Panel Practitioners—Inviting Epidemics to London—Sanatorium Stars—A Curious Point in Sanitary Law—The Treatment of Adenoids at Liverpool—The Medical Examination of Recruits—Some Tit-Bits in a Hospital "Gazette"—The Sculpture of Faces in Plasticine—The "Southern Cross" in May—Who Edits War Hospital Gazettes?—The Nurse Sub-Editor—1 Man 150 Votes—Lamp Day—An Unfortunate Day at Dudley—Soldiers and the Drug Habit—Prescriptions for Hospital Out-Patients—False Economies—The Way to Deal with a Critic—The Poor Law and Discharged Soldiers—A Reduction of Salary and a Protest—This Week's Drug Market. | |
| **Gas in Military Mines** ... ... ... ... ... | 169 |
| The Symptoms of Carbon Monoxide Poisoning. | |
| **A Medical Causerie** ... ... ... ... ... ... | 170 |
| **The Institutional Treatment of Consumption** | 171 |
| II.—Routine Time-Tables and Diet-Scales. | |
| **Remodelling a Hospital Garden** ... ... ... | 172 |
| A Practical Example at Hertford. | |
| **National Insurance Act Developments** ... ... | 173 |
| A Decision of the Court of Appeal and its Effects on the Right to Benefits. | |
| **Passing Events and Topics** ... ... ... ... ... | 174 |
| **The Editor's Letter-Box** ... ... ... ... ... | 175 |
| The "Artistic Temperament": The Real Artist's Temperament Analysed. | |
| Charing Cross Hospital. | |
| **Hospital Results in 1915** ... ... ... ... ... | 176 |
| **The Matrons' and Sisters' Department** ... | 177 |
| III.—Nursing in the United States. | |
| General Effect of Registration. | |
| A Desecration of Florence Nightingale's Statue. | |
| Round the Hospitals. | |
| Some Agitators' Follies—Convicted of Grave Ignorance—Pretensions not Knowledge—Canny Scottish Nurses—Diligent Sisters' Prizes. | |
| **The College of Nursing, Limited** ... ... ... | 179 |
| A Great Meeting in Edinburgh. | |
| **Personalia** ... ... ... ... ... ... ... ... ... | 180 |
| **Medical Appointments** ... ... ... ... ... | 180 |
| **Annual Meetings** ... ... ... ... ... ... | 180 |
| **Matrons' and Sisters' Appointments** ... ... ... | 180 |
| **Parliament and the Profession** ... ... ... | iv |
| **Gifts to Hospitals** ... ... ... ... ... ... | iv |
| **The Institutional Worker** ... ... (Suppl.) | 1 |
| War Work at the Voluntary Hospitals. | |

